# Structure of a BAG6 (Bcl-2-associated Athanogene 6)-Ubl4a (Ubiquitin-like Protein 4a) Complex Reveals a Novel Binding Interface That Functions in Tail-anchored Protein Biogenesis*

**DOI:** 10.1074/jbc.M114.631804

**Published:** 2015-02-20

**Authors:** Naoyuki Kuwabara, Ryosuke Minami, Naoto Yokota, Hirofumi Matsumoto, Toshiya Senda, Hiroyuki Kawahara, Ryuichi Kato

**Affiliations:** From the ‡ Structural Biology Research Center, Photon Factory, Institute of Materials Structure Science, High Energy Accelerator Research Organization (KEK), 1-1 Oho, Tsukuba, Ibaraki 305-0801, Japan and; the § Department of Biological Sciences, Tokyo Metropolitan University, 1-1 Minami-Osawa, Hachioji, Tokyo 192-0397, Japan

**Keywords:** Membrane Protein, Protein Assembly, Small-angle X-ray Scattering (SAXS), Ubiquitin, X-ray Crystallography, Tail Anchor

## Abstract

BAG6 is an essential protein that functions in two distinct biological pathways, ubiquitin-mediated protein degradation of defective polypeptides and tail-anchored (TA) transmembrane protein biogenesis in mammals, although its structural and functional properties remain unknown. We solved a crystal structure of the C-terminal heterodimerization domains of BAG6 and Ubl4a and characterized their interaction biochemically. Unexpectedly, the specificity and structure of the C terminus of BAG6, which was previously classified as a BAG domain, were completely distinct from those of the canonical BAG domain. Furthermore, the tight association of BAG6 and Ubl4a resulted in modulation of Ubl4a protein stability in cells. Therefore, we propose to designate the Ubl4a-binding region of BAG6 as the novel BAG-similar (BAGS) domain. The structure of Ubl4a, which interacts with BAG6, is similar to the yeast homologue Get5, which forms a homodimer. These observations indicate that the BAGS domain of BAG6 promotes the TA protein biogenesis pathway in mammals by the interaction with Ubl4a.

## Teaser

**Background:** Both BAG6 and Ubl4a are mediators of tail-anchored (TA) protein biogenesis and protein quality control.

**Results:** The crystal structure of the BAG6-Ubl4a heterodimerization domain reveals a unique binding interface.

**Conclusion:** The BAG-similar domain of BAG6 is completely distinct from other canonical BAG domains.

**Significance:** Ubl4a forms a complex with BAG6, whereas its yeast homologue Get5 forms a homodimer.

## Introduction

Co-chaperones of the Bcl-2-associated athanogene (BAG)^3^ family are characterized by the presence of a BAG domain, which is a conserved region that mediates direct interactions with the ATPase domains of molecular chaperones (1–4). Six BAG family members have been reported to date in humans; these proteins regulate, both positively and negatively, the functions of Hsp70/Hsc70 (4). BAG6/BAT3/Scythe (5) is considered to be a member of the BAG family because it contains a sequence homologous to the BAG domain at its C terminus and indeed collaborates with Hsp70 family molecular chaperones (4, 6–9). Recently, BAG6 was identified as an essential factor in the ubiquitin-mediated metabolism of newly synthesized defective polypeptides (10–14). BAG6 physically interacts with aggregation-prone polypeptides and regulates their proteasomal degradation (11). BAG6 possesses intrinsic affinity for exposed hydrophobicity of client proteins and modulates the dynamics of ubiquitin-positive cytoplasmic inclusions, such as aggresomes (11). Thus, previous observations shed light on the crucial role of BAG6 in the quality control of misfolded and/or mislocalized defective proteins (7, 8, 11–13, 15).

Recent work showed that BAG6 is also a mediator of tail-anchored (TA) transmembrane protein biogenesis (12, 16–18). TA proteins are a family of biologically important transmembrane domain polypeptides, including the endoplasmic reticulum translocon component Sec61β and the membrane-integrated SNARE proteins syntaxin and synaptobrevin. In the TA protein assembly pathway, BAG6 holds the hydrophobic transmembrane domain of newly synthesized TA proteins to maintain them in an unfolded but soluble state in the cytosol (12, 17). BAG6 then hands off the TA proteins to the central transmembrane recognition complex (TRC)/guided entry of TA proteins (GET) pathway component TRC40/Get3 for insertion into the endoplasmic reticulum membrane (12, 16, 17). Should assembly fail, mislocalized TA proteins are consigned to the ubiquitin-mediated degradation pathway via BAG6 (12, 13). A series of studies showed that BAG6 captures and shields the hydrophobic transmembrane domain of TA proteins just after their ribosomal release and determines whether they are assembled or degraded (7, 8, 12, 13, 17, 19).

In yeast, the GET pathway components are important for TA protein biogenesis (20–22). Hegde and colleagues (17) also identified the BAG6-binding proteins TRC35 and Ubl4a in mammals, which are homologs of the yeast GET pathway components Get4 and Get5, respectively. Get4 and Get5 form a stable heterodimer in yeast, and the UBL domain of Get5 interacts with the tetratricopeptide repeat-containing protein Sgt2 with the cytosolic molecular chaperone to recruit TA proteins (23–27). TA proteins are then handed off to the downstream ATPase Get3 via the direct Get4-Get5 interaction mediated by the N terminus of Get5, the essential domain for Get4 binding (25, 28). However, the mammalian counterpart of Ubl4a lacks the entire TRC35/Get4 binding site at its N terminus; therefore, Ubl4a cannot interact directly with TRC35 (26). Thus, the mechanisms for substrate transfer from Ubl4a to TRC40 in higher eukaryotes remain completely unknown. A recent crystal structure of Get5 revealed that the C terminus contains a homodimerization motif. Chartron *et al.* (28) pointed out that there are weak similarities between the C terminus of human Ubl4a and the homodimerization domain of yeast Get5, although mammalian Ubl4a never dimerizes by itself; consequently, the function of the C-terminal half of mammalian Ubl4a had remained mysterious. In addition, the yeast GET complex cannot be identical to its mammalian counterpart, because the yeast genome does not contain a gene homologous to *BAG6*. So far, nothing is known regarding the mechanism of substrate transfer or the physical interactions between the mammalian components of the Get-TRC-BAG6 complex.

In this study, we obtained evidence that the C terminus of BAG6 (previously designated as a BAG domain) is essential for tethering the C-terminal conserved stretch of Ubl4a, designated here as the TUGS (tethering Ubl4a to BAGS) domain. Subsequently, we determined the crystal structure of the C terminus of BAG6 and Ubl4a-TUGS complex. Unexpectedly, we found that the specificity and structure of C-terminal domain of BAG6 were found to be completely distinct from those of the canonical BAG domain structure. In agreement with this observation, the primary sequence of the BAG6 C terminus lacks some of the essential residues conserved within the BAG domain family. Therefore, we propose to designate the Ubl4a-binding region of BAG6 as a BAG-similar (BAGS) domain. Because both BAG6 and Ubl4a are mediators of TA protein biogenesis and protein quality control (17, 29), our identification of the BAGS-TUGS association provides novel insights into the function of this complex, which is dedicated to protein biogenesis as well as degradation events.

## EXPERIMENTAL PROCEDURES

### 

#### 

##### Peptide Mass Fingerprinting (PMF) Analysis for Xenopus Scythe/BAG6

Synthetic mRNA encoding C-terminally S-epitope (KETAAAKFERQHMDS)-tagged *Xenopus* BAG6 (Scythe) was microinjected into the 2-cell stage *Xenopus* embryos. Eighteen hours after injection, developing embryos were lysed with immunoprecipitation buffer (20 mm Tris-HCl, pH 7.5, 150 mm NaCl, 1 mm EDTA, 1% Nonidet P-40, 5 μg/ml leupeptin, 1 μg/ml pepstatin, 2 μg/ml aprotinin, 1 mm dithiothreitol), and Scythe-interacting proteins were affinity-purified using S-protein-agarose beads. Precipitates were subjected to SDS-PAGE and PMF analysis. PMF was performed basically as described by Minami *et al.* (10). Trypsin-digested peptides were desalted using a ZipTip-C18μ (Millipore) and mixed with 5 mg/ml α-cyano-4-hydroxycinnamic acid (Nacalai Tesque) in 0.1% trifluoroacetic acid and 50% (v/v) acetonitrile. The PMFs of proteins were obtained by MALDI-TOF/TOF MS on Ultraflex (Bruker Daltonics). A database search was performed using Mascot.

##### Constructs

The full-length *Bag6* cDNA of *Mus musculus* was amplified by PCR from an NIH3T3 cDNA library. The full-length cDNAs encoding Ubl4a, BAG1, BAG2, BAG5, and the BAG domain cDNAs for BAG3 and BAG4 of *Homo sapiens* were amplified by PCR from a HeLa cDNA library. The PCR products were subcloned into the pCI-neo expression vectors, whose products were fused with N-terminal 2S-tag (MKETAAAKFERQHMDSKETAAAKFERQHMDS) or 3FLAG tag (MDYKDHDGDYKDHDIDYKDDDDK). The truncated and mutated versions of BAG6 and Ubl4a were prepared by PCR, using pCI-neo-based vectors as templates.

##### Protein Expression and Purification

HeLa cells were cultured in Dulbecco's modified Eagle's medium (Sigma) supplemented with 10% heat-inactivated calf serum at 37 °C under a 5% CO_2_ atmosphere. DNA transfection was performed using Lipofectamine 2000 (Life Technologies, Inc.). For expression of GST-fused proteins, *Escherichia coli* BL21(DE3) cells were used with pGEX6P1-based expression vectors. To isolate GST-tagged proteins, *E. coli* extracts were applied to glutathione-immobilized Sepharose beads (GE Healthcare). The beads were extensively washed with buffer B (20 mm Tris-HCl, pH 7.5, 1 mm EDTA, 150 mm NaCl, 5% glycerol, and 0.1% Triton X-100) and eluted with 30 mm glutathione in buffer B. The GST fusion proteins thus obtained were dialyzed against dialysis buffer (20 mm Tris-HCl pH 7.5, 150 mm NaCl, and 10% glycerol). In some cases, the GST tag was cleaved with PreScission protease (GE Healthcare) and used for analysis.

##### Immunoprecipitation and Western Blotting

**FIGURE 1.**
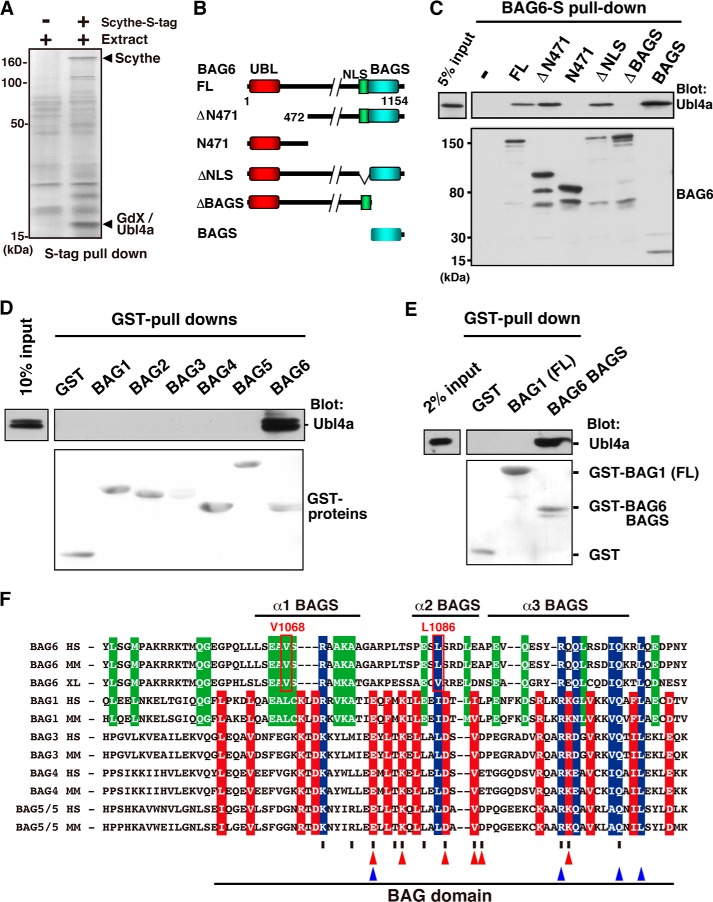
**The BAGS domain of BAG6 is a specific binding site for Ubl4a.** *A*, Scythe/BAG6-associated proteins in *Xenopus* embryos identified by PMF analysis. Precipitates obtained with S-tagged Scythe were subjected to SDS-PAGE, and the gel was silver-stained. *B*, schematic representation of the BAG6-deletion mutant proteins used in this study. *Numbers* denote corresponding amino acids. *C*, Ubl4a binding assay with a series of BAG6 deletion mutants. Full-length (*FL*) 2S-tagged BAG6 and its truncated derivatives were expressed in HeLa cells. Each form of BAG6 was affinity-purified using S-protein-agarose beads. After washing the beads, recombinant FLAG-tagged Ubl4a was mixed and precipitated, and bound materials were immunoblotted with anti-Ubl4a and anti-S-peptide antibodies. The BAGS domain of BAG6 is necessary and sufficient for Ubl4a binding. *D* and *E*, bacterially produced GST-tagged BAG domains derived from BAG1, BAG2, BAG3, BAG4, or BAG5 or the BAGS domains of BAG6 were mixed with purified FLAG-tagged Ubl4a *in vitro*. After GST pull-down, the precipitates were probed with an anti-FLAG antibody to detect Ubl4a co-precipitation (*top*). Precipitated GST proteins were stained with Ponceau S (*bottom*). Full-length BAG1 (*FL*) was also examined. *F*, multiple amino acid sequence alignments of the BAGS domain of BAG6 and BAG domains of BAG1, BAG3, BAG4, and BAG5. We exclude BAG2 from this alignment because of its extremely low amino acid conservation relative to other BAG family proteins. *Red box*, conserved residues in all BAG family proteins except for BAG6. *Green box*, conserved residues between BAG1 and BAG6 (showing validity of BAG1-BAG6 alignment). *Blue box*, conserved residues in all of these proteins. More than half of the residues reported to be involved in the Hsp70 interaction in BAG1 are not conserved in the BAG6 BAGS domain (shown as *red triangles*). *Vertical slash*, residues in human BAG1 that interact with Hsp70 (defined by Briknarova *et al.* (53)). *Blue triangles*, four strictly conserved residues in BAG family proteins (defined by Thress *et al.* (6)). Three α-helical regions of the BAGS domain are indicated by *bars. HS*, *H. sapiens*; *MM*, *M. musculus*; *XL*, *Xenopus laevis*.

HeLa cells were washed with ice-cold phosphate-buffered saline and lysed with buffer A (20 mm Tris-HCl, pH 7.5, 5 mm EDTA, 150 mm NaCl, 5% glycerol, and 1% Nonidet P-40). The lysates were sonicated for 1 s and centrifuged at 20,000 × *g* for 20 min at 4 °C, and the resulting supernatant was incubated with anti-FLAG M2-agarose (Sigma) for 2 h at 4 °C. After the beads were washed four times in buffer A, the purified complexes were eluted in SDS sample buffer. In the case of Fig. 1*C*, the cells were lysed with radioimmune precipitation assay buffer (50 mm Tris-HCl, pH 7.5, 150 mm NaCl, 0.1% SDS, 0.5% sodium deoxycholate, and 1% Nonidet P-40). The supernatant was incubated for 2 h at 4 °C with 3 μl of S-protein-agarose beads (Novagen). After the beads were washed four times with radioimmune precipitation assay buffer, they were incubated with recombinant Ubl4a protein in buffer A. After the beads were washed four times with buffer A, bound materials were eluted with SDS sample buffer. For Western blotting, whole-cell lysates and immunoprecipitated materials were separated by SDS-PAGE and transferred onto nitrocellulose membranes (Bio-Rad). The membranes were then immunoblotted with the indicated specific antibodies. The antibody against BAG6/Scythe was obtained as described previously (11). To prepare antiserum against Ubl4a, a rabbit was immunized with recombinant full-length Ubl4a. The following antibodies were purchased and used for immunological analyses: anti-FLAG tag (Sigma), anti-S-peptide (Santa Cruz Biotechnology, Inc.), anti-β-actin (Sigma), and anti-GST (Santa Cruz Biotechnology).

##### GST Pull-down Assay

GST-tagged proteins were incubated with 4 μl of glutathione-immobilized Sepharose beads, and the beads were washed four times with buffer B. GST-tagged protein beads were mixed with the indicated proteins, and the co-precipitated materials were analyzed by immunoblot experiments.

##### Surface Plasmon Resonance (SPR) Measurement

SPR binding assays were performed at 298 K on a Biacore T-200 system (GE Healthcare). HBS-P buffer (10 mm HEPES, 150 mm NaCl, 0.05% (v/v) Surfactant P20, pH 7.2) was used as a the running buffer at a flow rate of 30 μl/min. BAGS domain (residues 1048–1123) was directly immobilized on a CM5 sensor chip by amine coupling. Wild-type and mutant Ubl4a were used as analytes. The net response was calculated by subtracting the background response from the binding response. The results were analyzed using the Biacore T-200 evaluation software (GE Healthcare). Sensorgram curves were curve-fitted to a two-state reaction model.

##### Circular Dichroism (CD) Analysis

CD spectra were recorded in 1-mm quartz cuvettes on a J-815 CD spectrometer (Jasco). All data were collected from 200 to 260 nm at a scanning speed of 50 nm/min at 293 K. Three scans were averaged for each sample. All proteins were used at a concentration of 100 μm. The α-helical content was calculated from the value at 222 nm (30). Secondary structure contents were predicted using the K2D3 software (31).

##### Crystallization

BAG6 BAGS (residues 1048–1123) and Ubl4a TUGS (residues 95–147) domains were co-expressed in *E. coli* using pGEX6P1 (BAGS domain) and pCOLA Duet (Ubl4a TUGS domain). Cell extract was purified on a GS4B (GE Healthcare) affinity column, and GST was removed by protease cleavage. The sample was further purified by MonoS (GE Healthcare) ion exchange chromatography. The eluent sample was loaded onto a Superdex-75 (GE Healthcare) gel filtration column pre-equilibrated with a solution of 10 mm HEPES-NaOH at pH 7.0, 100 mm NaCl, and 1 mm DTT. The purified protein was then concentrated to 10–15 mg/ml.

Purified BAGS domain-TUGS domain complex was subjected to initial crystallization screening using an automated protein crystallization and monitoring system (32). After that, optimization of conditions was carried out by a hanging drop vapor diffusion procedure. Finally, rod-shaped crystals were obtained by mixing 0.9 μl of 7.5 mg/ml purified protein solution, 0.9 μl of reservoir solution (0.1 m CAPS-NaOH (pH 9.6), 1.9–2.0 m AmSO_4_, 0.2 m LiSO_4_, and 4% 2,2,2-trifluoroethanol) and 0.2 μl of 4 mm CHAPS (Hampton Research) and equilibrated with 400 μl of the reservoir solution at 293 K. For phase determination, iodide-derivative crystals were prepared by soaking in reservoir solution containing 0.5 m NaI for 1 h at room temperature.

##### X-ray Data Collection and Crystallographic Analysis

Prior to data collection, crystals were soaked in reservoir solution containing 21% xylitol and 12% ethylene glycol for cryoprotection and then frozen in a stream of nitrogen gas at 100 K. Native diffraction data and iodide-derivative data were collected at BL-17A (Photon Factory at the High Energy Accelerator Research Organization, Tsukuba, Japan). All diffraction data were first evaluated using the PF remote data monitoring system (PReMo) and processed using XDS (33). A single anomalous dispersion dataset was collected from NaI-soaked crystals. Seven iodide sites were found, and phases were estimated using Phenix.autosol. The initial figure of merit was 0.39, and the value changed to 0.66 after density modification. Initial model building was performed by Phenix.autobuild (34). Further model building and refinement were performed with COOT (35) and Phenix.refine (36), respectively. Diffraction and refinement data statistics are summarized in Table 1.

**TABLE 1:** **Data collection and refinement statistics** Statistics for the highest resolution shell are shown in parentheses.

	Native	Iodide-soaked crystal
**Data collection**		
Beamline	PF BL1A	PF BL17A
Wavelength (Å)	1.100	1.600
Resolution (Å)	48.48-1.85 (1.89-1.85)	47.87-2.39 (2.48-2.39)
Space group	*P*6_2_	*P*6_2_
Unit cell (Å)	*a* = *b* = 72.99, *c* = 48.48	*a* = *b* = 71.97, *c* = 47.84
No. of unique reflections	12,699	5701
Multiplicity	15.2	10.8
Completeness (%)	100.0 (100.0)	100.0 (100.0)
*R*_merge_ (%)	4.5 (349.1)	12.9 (160.7)
Mean *I*/σ*I*	31.6 (1.1)	14.9 (1.6)
Wilson *B*-factor	46.5	53.9
Mean (*I*) half-set correlation (CC half)	1.000 (0.530)	0.998 (0.628)

**Refinement**		
No. of complex molecules in the asymmetric unit	1	
No. of non-hydrogen atoms	901	
Macromolecules	828	
Ligands	52	
Water	21	
Root mean square deviation from ideal value		
Bond angles (degrees)	1.1	
Bond length (Å)	0.007	
*R*_work_ (%)	20.4	
*R*_free_ (%)	23.7	
Ramachandran plot		
Favored (%)	98	
Outlier (%)	0	
Average *B*-factor	68.1	
Proteins	66.5	
Ligand	99.7	
Solvent	56.3	

##### Small Angle X-ray Scattering (SAXS) Analysis

SAXS experiments were performed at BL-10C (Photon Factory at the High Energy Accelerator Research Organization, Tsukuba, Japan). The sample-to-detector distance was 1 m, and the detector was a PILATUS 300KW (Dectris). The distance and beam center were calibrated by powder diffraction of AgBh using the FIT2D software (37). Circular averaging was performed with FIT2D (38). Before measurements, sample buffer was exchanged with 10 mm HEPES-NaOH (pH 7.0), 150 mm NaCl, and 2 mm DTT by dialysis. X-ray scattering data were collected for each sample at five different protein concentrations (1.1–5.3 mg/ml for BAGS-TUGS complexes and 0.85–1.8 mg/ml for BAGS-full-length Ubl4a complexes) with five successive x-ray exposure times of 1 min. Circular averaging was performed with FIT2D, and further analyses of scattering intensity profiles were performed with PRIMUS from the ATSAS software package (39). The zero-angle scattering intensity, *I*(0), and radius of gyration, *R_g_*, were calculated using the Guinier approximation. The *I*(0) and *R_g_* of each protein complex were determined by extrapolation to zero protein concentration. The molecular masses were estimated from *I*(0) of ovalbumin (Sigma), used as a standard. The pair-distance distribution function, *P*(*r*), was calculated by the indirect Fourier transform method using GNOM (40). Ten independent dummy residue models were made with the *P*(*r*) functions using GASBOR (41), and these models were aligned, averaged, and filtered using DAMAVER (42). All models were generated by assuming P1 symmetry. Rigid body docking of the SAXS model and crystal structures was carried out using SUPCOMB (43) and manually.

##### Cycloheximide (CHX) Chase Experiments

HeLa cells expressing FLAG-tagged Ubl4a were treated with the protein synthesis inhibitor CHX at a concentration of 10 μg/ml for the indicated periods. Whole-cell extracts were prepared at the indicated times after CHX addition and subjected to Western blot analysis with anti-FLAG antibody for detection of Ubl4a and its derivative proteins. Actin was used as a loading control.

## RESULTS

### 

#### 

##### The BAGS Domain of BAG6 Interacts with the C-terminal Domain of Ubl4a

Scythe is a *Xenopus* ortholog of mammalian BAG6 that regulates embryonic cell death (6), possibly by modulating ubiquitin-mediated protein degradation (10, 44). To elucidate the regulatory network, we investigated Scythe-binding factors in *Xenopus* egg extracts (10). Subsequently, we found that the ubiquitin-like (UBL) domain protein *Xenopus* GdX, which is homologous to mammalian Ubl4a and yeast Get5, co-precipitated with Scythe (Fig. 1*A*). Ubl4a, a binding partner of mammalian BAG6, is involved in the correct assembly of the TA protein Sec61β (12, 17).

To identify the Ubl4a-binding site within BAG6 in mammals, we prepared a series of 2S-tagged BAG6 deletion mutants (Fig. 1*B*) and examined their interaction with FLAG-Ubl4a in HeLa cells. 2S-tagged BAG6 co-precipitated with Ubl4a, and the C-terminal BAGS domain (previously identified as BAG) was sufficient for Ubl4a binding (Fig. 1*C*). Consistent with this result, a fragment encoding only this C-terminal BAGS domain co-precipitated with Ubl4a as efficiently as the full-length form of BAG6 (Fig. 1*C*). These results indicate that the BAGS domain of BAG6 is necessary and sufficient for binding to Ubl4a.

To examine the direct interaction between Ubl4a and the BAG6 BAGS domain, we purified recombinant proteins expressed in *E. coli* and performed GST pull-down analysis (Fig. 1*D*). The GST-BAGS domain of BAG6 co-precipitated with full-length Ubl4a, clearly indicating that BAG6 and Ubl4a directly interact with each other. Moreover, Ubl4a was recognized exclusively by the BAGS domain of BAG6, whereas BAG domains derived from BAG1, BAG2, BAG3, BAG4, and BAG5 (Fig. 1*D*), as well as the full-length form of BAG1 (Fig. 1*E*), failed to interact with Ubl4a. These results suggest that the BAG6 BAGS domain is distinct from other canonical BAG family members.

The BAG domain is widely believed to be an Hsp70-binding motif (2, 3). Although our *in vivo* binding assay confirmed that the BAG6 BAGS domain indeed co-precipitated with Hsp70 (6, 45), the interaction was much less efficient than those of other BAG domains.^4^ In accordance with this observation, an amino acid alignment of BAG domains revealed that the primary sequence of the BAG6 BAGS domain is distinct from those of other BAG domain family proteins (Fig. 1*F*). In particular, residues that interact with heat shock proteins (46), which are indicated by *vertical slashes* and *red triangles*, are poorly conserved in the BAG6 family, supporting the idea that the BAG6 BAGS domain is unique, functionally and at the sequence level, relative to other BAG family proteins (Fig. 1, *D–F*). Based on these observations, we speculate that the C-terminal region of BAG6 is specifically optimized for direct binding with Ubl4a and distinct from the previously defined canonical BAG domain.

##### The C-terminal Conserved Region of Ubl4a Is Essential for Its Interaction with the BAG6 BAGS Domain

**FIGURE 2.**
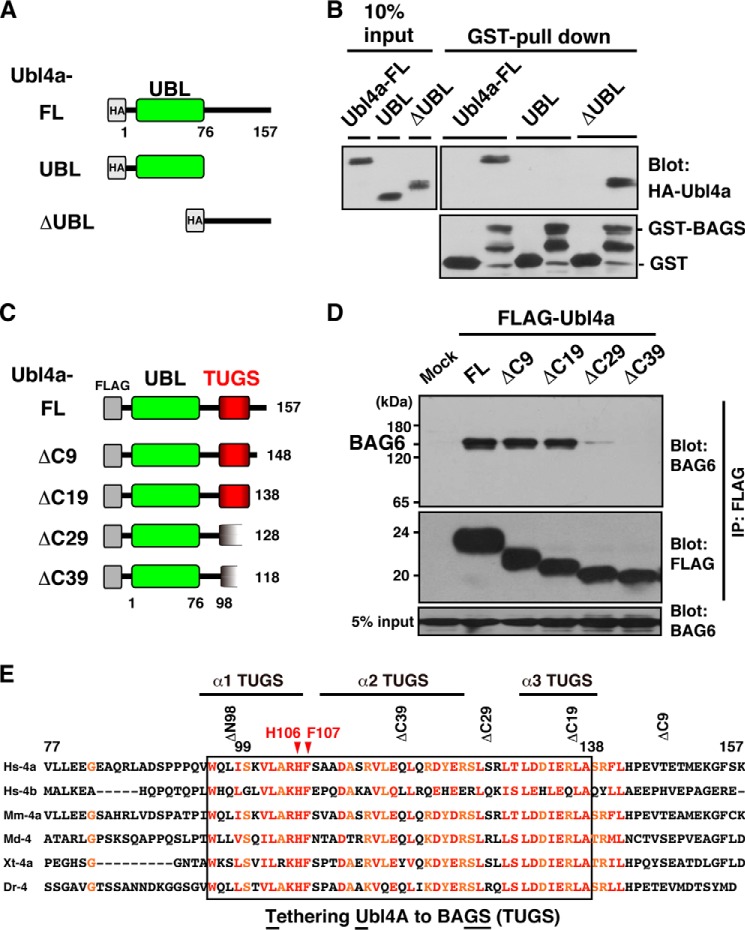
**The C-terminal TUGS-sequence of Ubl4a is essential for BAG6 binding.** *A* and *C*, schematic representation of full-length (*FL*) and a series of Ubl4a deletion mutants used in this study. *B*, binding assay of Ubl4a deletion mutants. The *E. coli*-expressed GST-BAG domain of BAG6 was incubated with a series of deletion mutants of purified 5HA-tagged Ubl4a. Material obtained by GST pull-down was probed with anti-HA (for Ubl4a) and anti-GST (for BAG6) antibodies. *D*, C-terminal deletion analysis of the minimal region of Ubl4a necessary for BAG6 interaction. A series of FLAG-tagged truncated Ubl4a were expressed in HeLa cells, and anti-FLAG immunoprecipitation was performed. Immunoprecipitates were then probed with anti-BAG6 antibody to examine their interaction. A 40-amino acid-long region (residues 99–138) of Ubl4a was identified as the sequence necessary for BAG6 interaction. *E*, sequence alignment of the C-terminal half (from the end of the UBL to the C terminus) of vertebrate Ubl4a family proteins revealed a highly conserved island (indicated by *red characters*). This region covers the entire stretch necessary for BAG6 binding (*boxed*), and hence we designated it the TUGS (tethering Ubl4a to BAGS) sequence. Three α-helical regions are indicated by *bars. Hs*, *H. sapiens*; *Mm*, *Mus musculus*; *Md*, *Monodelphis domestica* (opossums); *Xt*, *Xenopus tropicalis*; *Dr*, *Danio rerio.*

Ubl4a was originally identified as a 157-amino acid housekeeping protein with a UBL domain in its N-terminal half (47). To identify the region of Ubl4a required for binding of the BAG6 BAGS domain, we prepared two Ubl4a deletion mutants (Fig. 2*A*). Deletion of the N-terminal UBL domain (residues 1–76) did not affect BAG6 binding, indicating that it is dispensable for the BAGS domain interaction (Fig. 2*B*). The C-terminal half of Ubl4a has no overall homology with known proteins in the database except for a recently suggested rudimentary homology to the yeast Get5 C terminus dimerization domain (28). As shown in Fig. 2*B*, the C-terminal half (residues 77–157) of Ubl4a (ΔUBL) is both necessary and sufficient for interaction with the BAG6 BAGS domain.

To determine the BAG6-binding region of Ubl4a more precisely, we prepared a series of truncation mutants (Fig. 2*C*). The C-terminal 19 residues of Ubl4a were dispensable for BAG6 interaction, whereas deletion of an additional 10 amino acids abolished the binding (Fig. 2*D*). In addition, the N-terminal 98 residues of Ubl4a are dispensable for the BAG6 interaction (data not shown). These experiments imply that an ∼40-amino acid stretch, from residue 99 to 138 of Ubl4a, includes residues critical for the BAG6 interaction (Fig. 2*E*). Sequence alignment of the C-terminal half of Ubl4a (and Ubl4b, which also binds BAG6)^4^ derived from various vertebrates revealed an evolutionarily conserved stretch (Fig. 2*E*, *red characters*) that covers the entire region necessary for BAG6 binding (Fig. 2*E*, *box*). Because this recognition sequence is essential for the BAG6 interaction, we designate this region as the TUGS (tethering Ubl4a to BAGS) domain.

##### Validation of the Interface between BAG6 and Ubl4a

**FIGURE 3.**
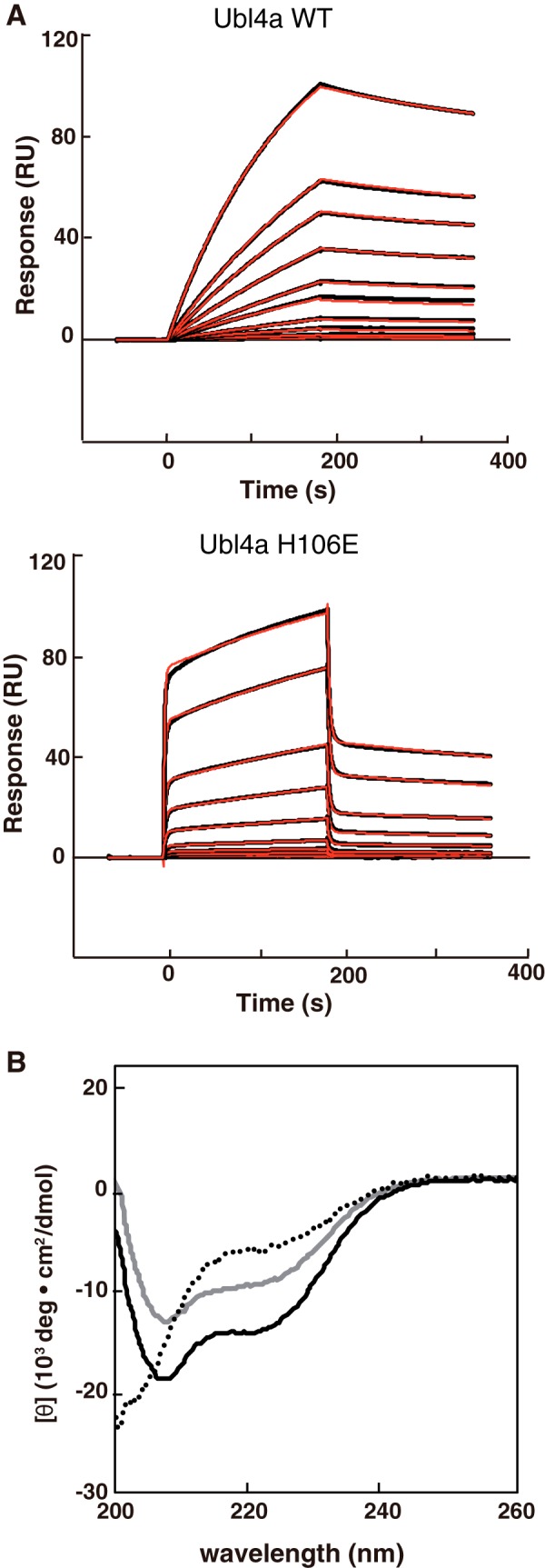
**Complex formation of the BAG6 BAGS domain and Ubl4a.** *A*, SPR analysis of binding of Ubl4a to immobilized BAG6 BAGS domain. SPR sensorgrams for various concentrations of full-length Ubl4a wild type (0.1, 0.2, 0.5, 1, 2, 3, 5, 7.5, 10, and 20 nm) and H106E mutant (0.01, 0.02, 0.05, 0.1, 0.2, 0.5, 1, 5, 10, 20, and 50 μm) are shown as *black curves*. A two-state reaction model was fitted to the data, and the resulting fit is shown in *red. B*, CD spectra of BAG6 BAGS domain (*dotted line*), full-length Ubl4a (*gray line*), and the complex (*black continuous line*) are shown. Concentrations of recombinant proteins were 100 μm each.

Using the bacterially expressed BAGS domain of BAG6 (residues 1048–1123) and the C-terminal region of Ubl4a (residues 95–147), we characterized the BAG6-Ubl4a interaction. To measure the affinity between BAG6 BAGS and Ubl4a TUGS, we carried out SPR experiments (Fig. 3*A*). Surprisingly, the calculated overall dissociation constant was very small, 2.2 ± 0.5 nm, suggesting that these two proteins form a very tight complex.

Next, we studied the secondary structures of the proteins in solution using CD measurements (Fig. 3*B*). The spectrum of Ubl4a exhibited large negative peaks around 209 and 222 nm, which indicated an extensive α-helical structure; the α-helical content was estimated as 24% from the value at 222 nm (30). On the other hand, the profile of BAG6 BAGS exhibited a large negative value at 200 nm but a small negative value at 222 nm, suggesting that it is almost unstructured; the α-helical content was estimated as 14% from the value at 222 nm. When BAG6 BAGS and Ubl4a were mixed, the profile revealed a typical α-helical structure whose intensity was greater than that of Ubl4a or BAG6 BAGS alone; the α-helical content was estimated to be 38% from the value at 222 nm. The secondary structure content was calculated using the K2D3 software (31). The α-helix and β-sheet contents were estimated to be 26 and 18% (Ubl4a), 19 and 16% (BAG6 BAGS), and 43 and 10% (BAG6 BAGS + Ubl4a), respectively. Thus, α-helical content increased upon complex formation. The change in β-sheet content was smaller than the change in α-helical content, and β-sheet contents were less than 20% in all cases. Thus, we suspect that the BAG6 BAGS fragment is disordered and/or non-helical in its monomeric form but forms helical structures upon Ubl4a binding.

##### BAG6-Ubl4a Forms a Heterodimer with Six α-Helices

**FIGURE 4.**
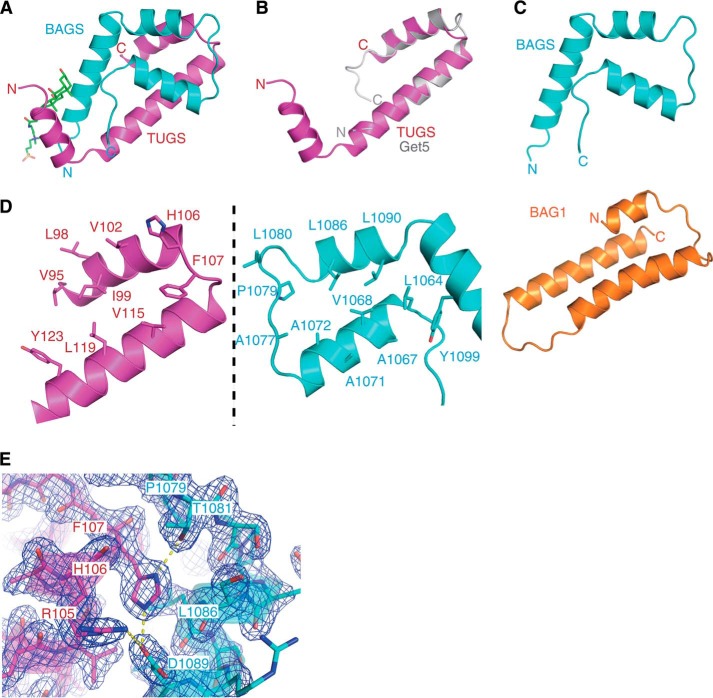
**Structure of the complex containing the TUGS region of Ubl4a and the BAG6 BAGS domain.** *A*, the overall structures of human Ubl4a and BAG6 are represented in a *ribbon model*. TUGS and BAGS domains are shown in *magenta* and *cyan*, respectively. Ubl4a TUGS consists of three helices named Ubl4a-α1 (α1TUGS), -α2 (α2TUGS), and -α3 (α3TUGS). BAG6 BAGS also consists of three helices named BAG6-α1 (α1BAGS), -α2 (α2BAGS), and -α3 (α3BAGS). One CHAPS molecule in the crystal structure is shown as a *green stick model. B*, structural comparison of the TUGS region of the BAGS-TUGS complex with the yeast Get5 C terminus homodimer (Protein Data Bank code 3VEJ, chain A). The root mean square deviation between the two models is 0.732 Å. TUGS is shown in *magenta*, and Get5 is shown in *gray. C*, comparison of the BAG6 BAGS domain and mouse BAG1 BAG domain (Protein Data Bank code 1I6Z). The homologous region of the mouse BAG1 domain (residues 135–196), according to the alignment in Fig. 1*F*, is presented as an *orange schematic model*. The BAG6 BAGS domain is shown in *cyan. D*, *open book presentation* of the hydrophobic interface of Ubl4a TUGS (*left*) and BAG6 BAGS (*right*) domains. The hydrophobic side chains, located on the interface, are shown as *stick models. E*, *close-up view* of the loop region between α1TUGS and α2BAGS. His-106 of Ubl4a TUGS makes hydrogen bonds with Thr-1089 (2.7 Å). Asp-1089 of BAG6 BAGS (2.7 Å), respectively. Asp-1089 of BAG6 BAGS also weakly interacts with Arg-105 of Ubl4a TUGS (3.5 Å) via a hydrogen bond. 2*F_o_* − *F_c_* omit map at contoured 1.5σ is shown in *blue*.

To determine the crystal structure of the BAG6 BAGS and Ubl4a TUGS complex, we co-expressed both proteins in *E. coli* and purified them as a complex. We subjected the sample solution to crystallization screening, optimized the conditions, and obtained crystals. The x-ray crystal structure of the complex was determined at 1.85 Å resolution. Unambiguous electron density allowed modeling of residues 1058–1112 of BAG6 BAGS and 94–143 of Ubl4a TUGS. The overall structure of the complex consists of six α-helices, three from BAG6 BAGS and three from Ubl4a TUGS (Fig. 4*A*).

The structure of the first (α1TUGS, residues 95–104) and the second (α2TUGS, residues 109–128) α-helices of Ubl4a TUGS is nearly identical to that of the C-terminal dimerization domain in yeast Get5 (28), despite the extensive divergence in the primary sequences, especially at the protein surfaces (Figs. 2*E* and 4*B*). Furthermore, Human Ubl4a (and the TUGS fragment itself) never self-dimerized, in clear contrast to the self-dimerization of Get5. The third α-helix (α3TUGS, residues 133–142) and the surrounding amino acid residues are well conserved (Fig. 2*E*); however, those residues are dispensable for BAG6 binding (Fig. 2*D*), and their significance remains unclear.

BAG6 BAGS consists of three α-helices, residues 1064–1075 (α1BAGS), 1083–1090 (α2BAGS), and 1093–1112 (α3BAGS) (Figs. 1*F* and 4*A*). The tertiary structure of BAG6 BAGS was quite distinct from that of the canonical BAG domain identified in BAG1, which adopts a three-helix bundle fold (Fig. 4*C*) (4). Given that several essential residues in the canonical BAG domain are substituted in the BAG6 BAGS domain (Fig. 1*F*), we propose that the BAG6 BAGS domain might be an evolutionally related but functionally distinct domain dedicated to the TUGS interaction.

α1BAGS crosses with α2TUGS forming at a 60° angle, and α2BAGS crosses with α1TUGS forming at a 20° angle, respectively, forming a four-α-helix bundle. The dimer interface primarily consists of the hydrophobic patch on BAG6 (Leu-1064, Val-1068, Ala-1071, and Ala-1072 in α1BAGS; Ala-1077, Pro-1079, and Leu-1080 in the loop region of α1BAGS and α2BAGS; Leu-1086, and Leu-1090 in α2BAGS; and Tyr-1099 in α3BAGS) that docks against Ubl4a (Val-95, Leu-98, Ile-99, and Val-102 in α1TUGS; Phe-107 in the loop region between α1TUGS and α2TUGS; and Val-115, Leu-119, and Tyr-123 in α2TUGS) (Fig. 4*D*). The hydrophobic residues in Get5 (Ile-182, Phe-190, Val-200, Leu-204, and Trp-208) that are reported to form the core for its homodimerization (28) are structurally and sequentially conserved in Ubl4a as Ile-99, Phe-107, Val-115, Leu-119, and Tyr-123.

There are close hydrophobic associations between the side chain of Phe-107 (Ubl4a) and those of Val-1068, Ala-1077, Pro-1079, and Leu-1086 of BAG6. Similarly, Val-102 of Ubl4a associates with Leu-1086, Asp-1089, and Leu-1090 of BAG6. Furthermore, there are several electrostatic (or hydrogen-bonding) interactions across the dimer. His-106 of Ubl4a forms electrostatic bridges with Thr-1081 (2.7 Å) and Asp-1089 (2.6 Å) of BAG6 (Fig. 4*E*), and all of these residues are conserved across vertebrates. In addition, the side chain of Ubl4a Tyr-123 also contacts Asp-1106 of BAG6 at a distance of 2.6 Å. One CHAPS molecule was visible in this crystal structure, and it interacted with α3TUGS and α3BAGS (Fig. 4*A*). This interaction was far from the BAGS-TUGS interaction site, but it was important for overcoming the crystal anisotropy.

##### Characterization of the Interface between BAG6 and Ubl4a

**FIGURE 5.**
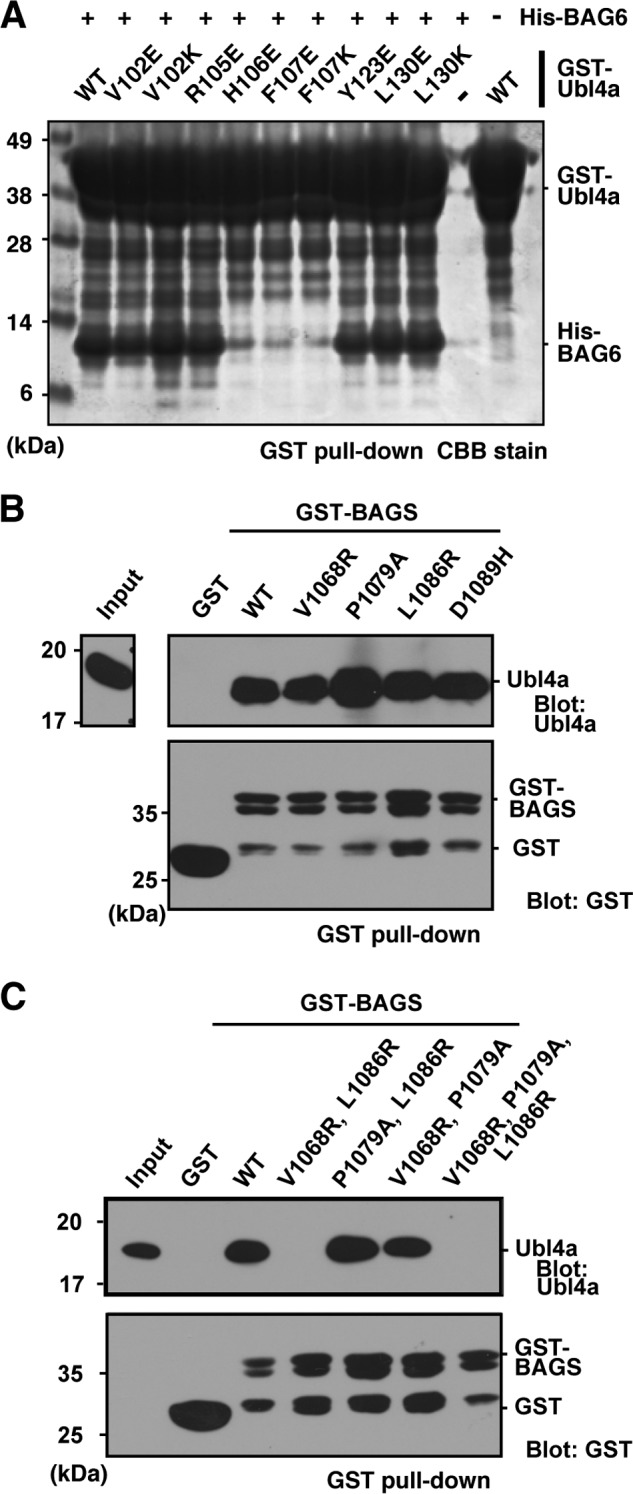
**Importance of conserved residues at the interface of the BAGS-TUGS interaction.** *A*, a series of bacterially expressed GST-tagged Ubl4a (full-length) variants containing point mutations were immobilized to glutathione-Sepharose 4B and then incubated with His-tagged BAGS domain fragment (residues 1048–1124). After the beads were washed several times, the samples were analyzed by SDS-PAGE, and precipitated proteins were detected by Coomassie Brilliant Blue (*CBB*) staining. GST-Ubl4a (40 kDa) and His-tagged BAG6 (10 kDa) are indicated. *B* and *C*, a series of GST-tagged BAGS domain mutants were incubated with full-length Ubl4a. GST precipitants were subjected to Western blotting with the indicated antibodies.

To confirm the putative interface of the complex and the importance of the amino acid residues found in our structure, we constructed a series of point mutant proteins of BAG6 BAGS and Ubl4a TUGS and analyzed their interaction. First, we introduced mutations into Ubl4a TUGS, and then we analyzed the interaction with wild-type BAG6. As shown in Fig. 5*A*, mutations of His-106 and Phe-107 resulted in drastically lower affinity for BAG6, indicating that the BAG6-Ubl4a interaction is influenced not only by hydrophobicity but also by electrostatics. No similar effect was seen in the other mutants we tested. Because His-106 and Phe-107 are located on the linker between α1TUGS and α2TUGS helices of Ubl4a, the linker region may be important for Ubl4a-BAG6 complex formation. To confirm the importance of the hydrophobic interaction between Phe-107 of Ubl4a TUGS and Val-1068, Pro-1079, and Leu-1086 of BAG6 BAGS, we constructed combinations of the BAG6 mutants. Single substitution of these residues had no apparent effect on their association, whereas the triple mutation (simultaneous substitutions of V1068R, P1079A, and L1086R) abolished the BAGS-TUGS interaction (Fig. 5*B*). Further analysis showed that double mutation (V1068R and L1086R) was sufficient to eliminate the association (Fig. 5*C*). These results indicate that both Val-1068 and Leu-1086 of BAG6 are critical for determining the binding affinity between BAG6 BAGS and Ubl4a TUGS.

##### BAG6-Ubl4a Complex Is Extended in Solution

**FIGURE 6.**
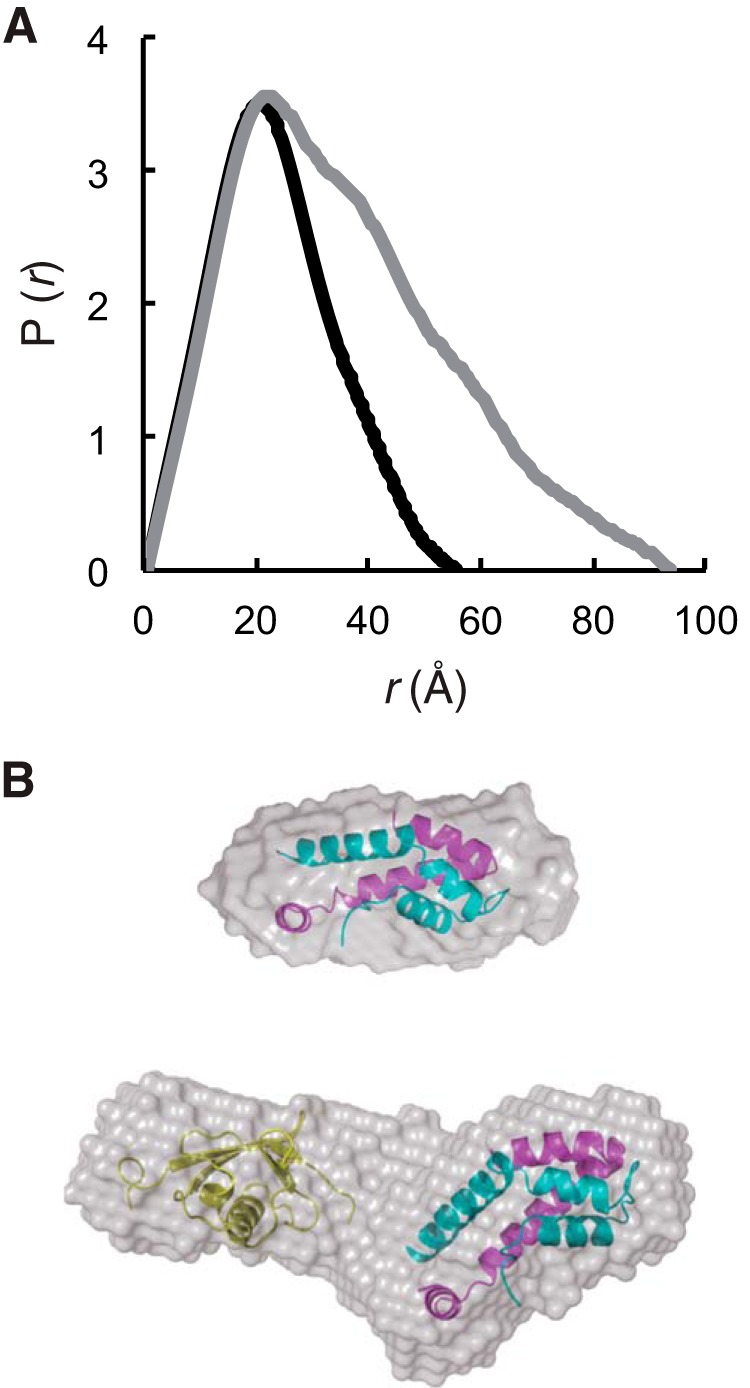
**BAGS-Ubl4a complex forms extended structure in solution.** *A*, pair-distance distribution (*P*(*r*)) functions of BAGS-TUGS (residues 95–147) and BAGS-Ubl4a (residues 1–157) complexes are shown by *black* and *gray lines*, respectively. These functions were calculated from the x-ray scattering intensity profile. *D*_max_ values of the complexes are 55 Å (BAGS-TUGS) and 93 Å (BAGS-Ubl4a), respectively. *B*, SAXS envelopes calculated using GASBOR, of the BAGS-TUGS complex (*top*) and BAGS-Ubl4a complex (*bottom*). Each GASBOR model is shown as a *gray surface model*. Rigid body docking of the BAGS-TUGS complex structural model into the BAGS-TUGS complex model using SUPCOMB is shown (*top*). In the case of the BAGS-Ubl4a complex, the NMR structure of Ubl4a UBL domain (Protein Data Bank code 2DZI) and the BAGS-TUGS complex structure were manually docked into the model (*bottom*).

The N-terminal UBL domain of Ubl4a is involved in recruitment of the co-chaperone SGTA with BAG6 (26, 27) and a C-terminal TUGS domain that binds with BAG6 (this study). Although high resolution crystal structures are now available for the BAG6 BAGS and Ubl4a TUGS complex, the solution structure is still important for elucidating the spatial arrangement. We collected SAXS data on the BAG6 BAGS (residues 1048–1123) and Ubl4a TUGS (residues 95–147) complex and also the full-length Ubl4a (residues 1–157) complex. Guinier analysis showed that BAGS and TUGS form a heterodimer in solution (Table 2), which coincides well with the crystal structure. The complex between BAGS and full-length Ubl4a also forms a dimer in solution. The pair-distance distribution (*P*(*r*)) functions and *ab initio* models from SAXS data revealed that the BAGS-full-length Ubl4a complex is more extended than the BAGS-TUGS complex (Fig. 6).

**TABLE 2:** **Radius of gyration and molecular weight of BAG6 BAGS and Ubl4a complex by solution x-ray scattering**

	*R_g_*	Molecular mass	Molecular mass calculated from amino acid sequence
	Å	*kDa*	*kDa*
BAG6(1048–1123) and Ubl4a(95–147) complex	17.7 ± 0.2	16.5 ± 0.3	14.8
BAG6(1048–1123) and Ubl4a(1–157) complex	25.3 ± 0.5	24.0 ± 0.5	26.4

##### BAG6 Association Is Necessary for Stability of Ubl4a

**FIGURE 7.**
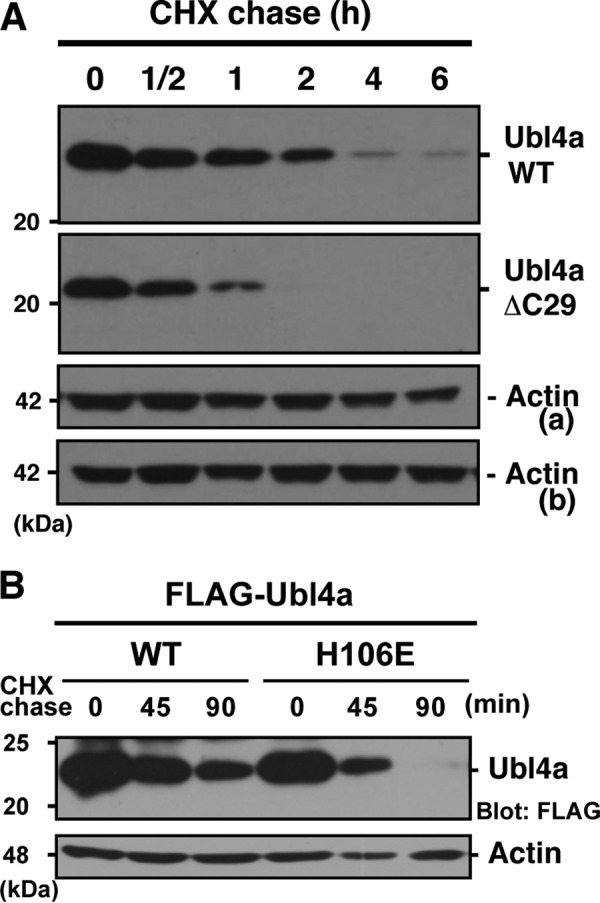
**BAG6 association stabilizes the Ubl4a protein.** *A*, Ubl4a protein is destabilized by loss of its C-terminal BAG6 binding region. Expression vectors encoding FLAG-tagged wild-type Ubl4a and its ΔC29 derivative were transfected into HeLa cells, and CHX chase analysis was performed, following Western blotting with anti-FLAG and anti-β-actin antibodies. Actin loading control for wild type (*a*) and for ΔC29 (*b*) are indicated. *B*, CHX chase analysis of wild-type and H106E mutant Ubl4a in HeLa cells.

The half-life of ectopically expressed wild-type Ubl4a in HeLa cells was 1.6 h (Fig. 7*A*). We found that a truncation mutant of Ubl4a lacking the C-terminal 29 residues (ΔC29), which lost the ability to bind BAG6, exhibited a decreased half-life of 0.6 h. This observation suggests that Ubl4a stability is governed by BAG6 binding. To analyze the relationship between the BAG6 association and the Ubl4a half-life, we utilized the H106E mutant of Ubl4a, which lost the ability to bind BAG6 in the pull-down experiment (Fig. 5*A*). We confirmed that H106E Ubl4a has reduced affinity for BAG6 in an SPR experiment (Fig. 3*A*). The calculated overall dissociation constant was 1.6 ± 0.08 μm, 3 orders of magnitude weaker than the wild type. Next, we examined the half-life of the BAG6-association defect mutant of H106E Ubl4a in HeLa cells and found that this mutant exhibited accelerated degradation (Fig. 7*B*), like the ΔC29 form of Ubl4a. This observation suggests that the tight interaction between Ubl4a TUGS and BAG6 BAGS determines the stability of Ubl4a. It remains a possibility that the mutations result in a shortened half-life for some other reason, such as destabilization of the structure in cells, although the purified proteins are stable *in vitro*.

## DISCUSSION

In budding yeast, GET proteins directly mediate the insertion of newly synthesized TA proteins into endoplasmic reticulum membranes. Similarly, mammalian BAG6, Ubl4a, and SGTA make up a trimeric complex that binds TA proteins post-translationally and then loads them onto the cytosolic ATPase TRC40, which in turn targets them to the endoplasmic reticulum (17, 19). A recent crystallographic analysis of yeast Get5, an ortholog of mammal Ubl4a, revealed that its C terminus contains a conserved homodimerization motif (28); however, Ubl4a does not engage in homodimeric interactions *in vivo* and *in vitro* (28).^4^ In this study, we showed that the C-terminal TUGS domain of Ubl4a is essential for BAG6 tethering. Given that BAG6 mediates oligomeric complex formation of Ubl4a, TRC35, and TRC40 (mammalian counterparts of Get5, Get4, and Get3, respectively), the C-terminal TUGS domain might be crucial for supporting BAG6-mediated Ubl4a-TRC35 complex formation in humans as an alternative to the direct Get5-Get4 interaction in yeast. Chartron *et al.* (25, 28) previously suggested that the Get5 N terminus, a unique Get4-binding domain in yeast, may be replaced in mammals by another protein(s) that could bridge between the Get4 homolog TRC35 and Ubl4a, and BAG6 is probably such a platform protein in mammals.

Little is known regarding the physical interactions of mammalian components of the BAG6-Ubl4a-TRC complex except for the recently identified Ubl4a-SGTA interaction (48, 49). The TUGS domain of Ubl4a by itself is a monomer, but its structure is similar to the dimerization motif found in yeast Get5 (28). Phe-107 of Ubl4a is structurally conserved in yeast Get5 as Phe-190, which is crucial for Get5 dimerization. On the other hand, His-106 of Ubl4a, which is not conserved in yeast Get5 either structurally or at the sequence level, is conserved in the BAG6 family. Because a point mutation changing His-106 to glutamine drastically reduced the affinity of Ubl4a for the BAGS domain, this residue might be critical for BAG6-Ubl4a heterodimer formation. Hydrophobic side chains of BAG6 BAGS are located on the sides of the helices and form the interaction. Because these hydrophobic residues are mostly conserved between vertebrates and fungi, BAG6 might have joined the TA protein assembly (GET) pathway by invading the Ubl4a/Get5 dimerization interface. The tight complex formation would be achieved by these interactions. Although His-106 faces toward Asp-1089 of the BAGS domain, substitution of Asp-1089 to a positively charged residue failed to abolish the interaction, suggesting that this electrostatic interaction is dispensable.

We used SAXS to show that the BAG6 BAGS-Ubl4a TUGS complex exists as a heterodimer in solution. The BAG6 BAGS-full-length Ubl4a complex is also a heterodimer with an elongated shape, suggesting that the N-terminal UBL of Ubl4a, where SGTA binds (26), extends on the opposite side of the BAGS-TUGS complex. Therefore, there should be no direct interaction between the N-terminal UBL and C-terminal TUGS of the BAG6-Ubl4a complex. Although the precise role of Ubl4a in protein quality control is currently obscure, Ubl4a might be a molecular gateway that recruits additional factor(s) to modulate protein quality control, such as chaperone machinery and/or ubiquitination enzymes, possibly through their N-terminal UBL domains. Indeed, the UBL domain of Ubl4a and its yeast homologue Get5 interacts with the tetratricopeptide repeat-containing protein SGTA/Sgt2, which forms a stable complex with the cytosolic molecular chaperone Hsp70/Ssa1p and the J-domain containing co-chaperone Hsp40/Ydj1 (23–27), thus recruiting a variety of BAG6 clients, including TA proteins, that expose hydrophobic residues (50, 51).

Ubl4a might be a non-covalent modifier that regulates the conformation of BAG6. Indeed, according to the CD results (Fig. 3*B*), the BAGS domain was disordered when expressed as a monomer, whereas a helical structure was induced by the association with Ubl4a. Conformational changes in BAG6 might facilitate its functions involved in recognition, holding, and release of substrates to their appropriate destinations. Ye and colleagues (48) also suggested that ubiquitination of the Ubl4a stimulates proteolytic cleavage of the BAG6 C terminus and causes endoplasmic reticulum-associated protein degradation inhibition. These results suggest that the BAG6 complex is involved in multiple regulatory pathways mediated by Ubl4a association.

Mammalian BAG6 is a 1,126-amino acid protein with a UBL domain in the N-terminal region and a BAGS domain in the C-terminal region. The N-terminal region is important for the protein quality control pathway, in particular for eliminating aggregation-prone polypeptides (11, 13). The C-terminal region, as we describe in this report, is a component of the human TA protein assembly pathway (12). Because SGTA binds to both UBL domains of BAG6 and Ubl4a (27, 28), BAG6 may work to bridge the two pathways through the high affinity to Ubl4a. Elucidation of the physiological significance of BAG6-Ubl4a complex is likely to provide important insights into the basic principles that could be exploited for protein biogenesis and degradation events.

## Footnotes

4 R. Minami and H. Kawahara, unpublished observations.

## Footnotes

3 The abbreviations used are:

BAG: Bcl-2-associated athanogene
CHX: cycloheximide
GET: guided entry of TA proteins
SAXS: small angle x-ray scattering
SPR: surface plasmon resonance
TA: tail-anchored
TUGS: tethering Ubl4a to BAGS
UBL: ubiquitin-like
TRC: transmembrane recognition complex
PMF: peptide mass fingerprinting
CAPS: 3-(cyclohexylamino)propanesulfonic acid.

## Addendum

After we submitted the manuscript, a similar complex structure of C-terminal domains of BAG6 and Ubl4a was reported (52).
